# Treatise on Analytic Nonlinear Optimal Guidance and Control Amplification of Strictly Analytic (Non-Numerical) Methods

**DOI:** 10.3389/frobt.2022.884669

**Published:** 2022-10-04

**Authors:** Timothy Sands

**Affiliations:** Sibley School of Mechanical and Aerospace Engineering, Cornell University, Ithaca, NY, United States

**Keywords:** optimization problems, control problems, nonlinear problems, mathematical modeling, transport theorem

## Abstract

Optimal control is seen by researchers from a different perspective than that from which the industry practitioners see it. Either type of user can easily become confounded when deciding which manner of optimal control should be used for guidance and control of mechanics. Such optimization methods are useful for autonomous navigation, guidance, and control, but their performance is hampered by noisy multi-sensor technologies and poorly modeled system equations, and real-time on-board utilization is generally computationally burdensome. Some methods proposed here use noisy sensor data to learn the optimal guidance and control solutions in real-time (online), where non-iterative instantiations are preferred to reduce computational burdens. This study aimed to highlight the efficacy and limitations of several common methods for optimizing guidance and control while proposing a few more, where all *methods are applied to the full, nonlinear, coupled equations of motion including cross-products of motion from the transport theorem*. *While the reviewed literature introduces quantitative studies that include parametric uncertainty in nonlinear terms, this article proposes accommodating such uncertainty with time-varying solutions to Hamiltonian systems of equations solved in real-time.* Five disparate types of optimum guidance and control algorithms are presented and compared to a classical benchmark. Comparative analysis is based on tracking errors (both states and rates), fuel usage, and computational burden. *Real-time optimization with singular switching plus nonlinear transport theorem decoupling is newly introduced and proves superior* by matching open-loop solutions to the constrained optimization problem (in terms of state and rate errors and fuel usage), while robustness is validated in the utilization of mixed, noisy state and rate sensors and uniformly varying mass and mass moments of inertia. *Compared to benchmark, state-of-the-art methods state tracking errors are reduced one-hundred ten percent. Rate tracking errors are reduced one-hundred thirteen percent. Control utilization (fuel) is reduced eighty-four percent, while computational burden is reduced ten percent, simultaneously, where the proposed methods have no control gains and no linearization.*

## 1 Introduction

**FIGURE 1:**
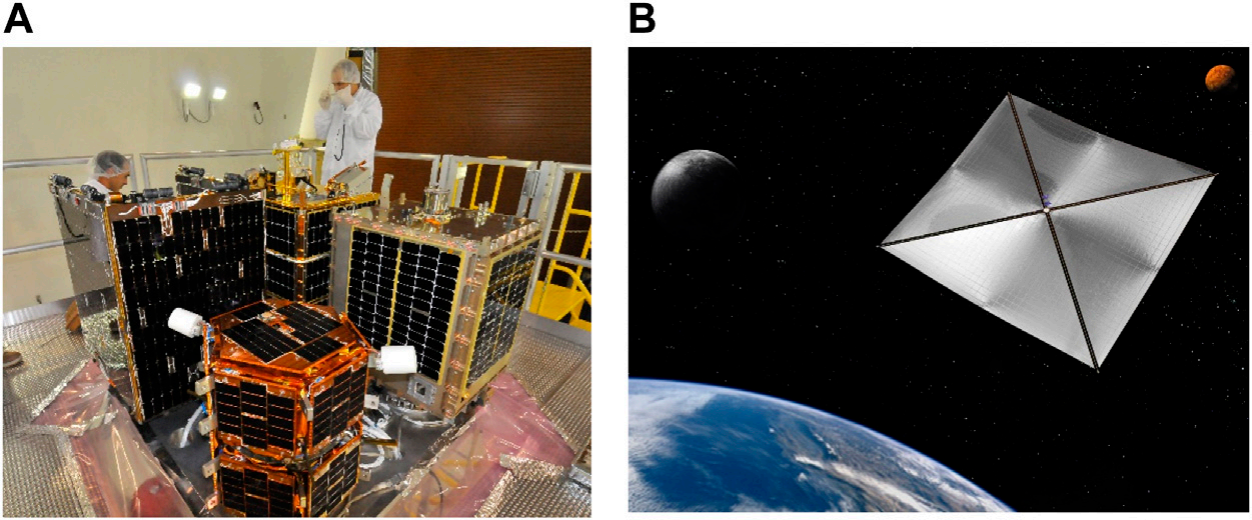
**(A)** NASA’s FASTSAT microsatellite readied to share ride to space (NASA, 2021a). **(B)** NASA ejects nanosatellite from microsatellite in space. Image used consistent with NASA policy, “NASA content (images, videos, and audios) are generally not copyrighted and may be used for educational or informational purposes without needing explicit permissions” (NASA, 2021b).

Considering intermittent coverage and communication delays with typical stellar satellites like those illustrated in Figure 1, autonomous guidance and control necessitates real-time on-board computation with demanding accuracy and robustness requirements, despite potentially coarsely known system characteristics, varying environmental conditions, and mission-related constraints. Many solutions have been developed, including optimal analytic methods for simple cases (Chai et al., 2019), while optimal methods for guiding and controlling realistic nonlinear systems ubiquitously necessitate either computational solutions or linearization to achieve analytical solutions. *This manuscript proposes new techniques for utilizing optimization techniques applied to the full, nonlinear, coupled equations of mechanical motion, and the techniques are analytic as opposed to numeric.* Rao proposed numerical trajectory optimization applied to orbital transfer problems (Rao et al., 2002) and also produced a survey of numerical methods for optimal control (Rao, 2009). Numerical methods are very quickly resorted to as researchers grapple with six nonlinear, coupled equations of mechanical motion (both translation and rotation). A generalized treatment method (again numerical) for optimization problems was proposed by Ross and Karpenko (2012) for such orbital transfer problems, spacecraft rendezvous and docking (Gao et al., 2009; Pontani and Conway, 2013; Bonnans and Festa, 2017), and planetary entry and hypersonic space planes (Windhorst et al., 1997; Arora, 2002; Chen et al., 2005; Ivanov et al., 2007; Zhang and Chen, 2011). Arguably, following the publication of Ross (2015), numerical optimization in general form realized the current dominance of numerical methods: for example, Tian et al. (2015) and Sagliano et al. (2017) for real-time (numerical) trajectory optimization and Chai et al. (2018a) and Chai et al. (2018b) for aero-assisted optimal tracking guidance.

Lacking ubiquitous analytic methods to treat the nonlinear, coupled systems of equations, linearization followed by least-squares optimization leads to the so-called Ricatti equations (NASA, 2021a) to produce optimal control gains (Kwakernaak and Sivan, 1972) with presumed error feedback (Kelly, 2005) in both continuous and discrete form (Flugge-Lotz, 1953). Optimization is sometimes sought after first implementing adaptive (Sands et al., 2009) methods to use feedback achieving predictability (Sands, 2019). Duprez et al. (2017) sought to tackle the nonlinear transport theorem terms by proposing control being a Lipschitz vector field on a fixed control set angular velocity, ω . This manuscript seeks to extend the notion of tacking nonlinear transport theorem to include time-varying angular velocities. Arguchintsev and Poplevko (2021) proposed an optimal control for linear hyperbolic systems of ordinary differential equations by estimating the residuals in terms of the value that characterizes the smallness of the measure of the domain of the needle variation of control. Emphasis was placed on problem formulation by Srochko et al. (2021), but the focus was parameterizing the cost functional rather than the nonlinear constraint function as done in this work.

Championed by Lorenz, physics-based methods (Sands and Ghadawala, 2011) were proposed to instantiate “self-sensing machines methods”, where the sensing functions are fully integrated on a drive to detect key operating characteristics including rotor position, torque, speed, temperature, and motor/load diagnostics” (Malecek, 2021). The physics-based methods codified optimal feedforward forms, which were later augmented with optimal feedback (Smeresky et al., 2020), instantiating the relatively new method referred to as deterministic artificial intelligence (D.A.I.). D.A.I. necessitates analytic forms of desired state trajectories (Baker et al., 2018) for the feedforward control and state observers (Heidlauf and Cooper, 2017; Willems, 1971) for the feedback control. In 2021, the utilization of Pontryagin’s approach (Pontryagin et al., 1962; Boltyanskii, 1971) to impose necessary conditions of optimality as a first step led to boundary-value problems that produce optimal controls, but *also* optimal trajectories (Sands, 2021) as alternatives to the sinusoidal trajectories recommended by Baker et al. (2018). These optimal trajectories are utilized in this manuscript as prescriptions for the coupled motion cross-products resulting from the inclusion of the transport theorem of motion.

Thus, the reader may consider the use of classical control methods (proportional plus velocity will be evaluated here) and seek to optimize control gains or use the Ricatti equation to seek linear-quadratic optimal classical control gains. Alternatively, time-optimal control may be considered a feedback form or control-minimizing control in an open-loop feedforward topology. Furthermore, real-time optimal controls could be derived that utilize feedback of state and rate in a matrix-inverse to enforce optimality in a closed-loop. Several different options for matrix inversion are available, generating subsets of the broader category of real-time optimal control. A key limitation of all the methods described so far is the inability to deal with nonlinear, coupled equations generated by the transport theorem in both translational and rotational mechanics.

Parametric uncertainty is another challenging aspect of nonlinear systems. Hu, et al. (2015) investigated nonlinear regression including parameter uncertainty estimates using the Monte Carlo and bootstrap methods to estimate nonlinear parameter uncertainties with a Microsoft Excel spreadsheet. Similarly, Monte Carlo statistical analysis is utilized here in MATLAB/SIMULINK and presented in section 3 Results. A modified James–Stein State Estimator (JSSE), named Modified James–Stein State Estimator (JSSE-M) was proposed by Meda-Campana (2018) as an alternative to filtering the states of nonlinear systems within a control scheme. Ferreres and Fromion (2010) studied the existence of limit-cycles in a closed-loop, which simultaneously contains nonlinearities and parametric uncertainties, addressed using three methods: 1) using a necessary condition of oscillation embodies in a graphical method, 2) checking the absence of limit-cycles despite parametric uncertainties using a sufficient condition of non-oscillation, and 3) using the necessary condition of oscillation to synthesize a controller which modifies the characteristics (magnitude and frequency) of the limit-cycle. A generically similar approach is used here, where necessary conditions of optimality are used to yield a nonlinear controller that can accommodate uncertainties in nonlinear systems. Arguably, a much more common approach to stability robustness of uncertain nonlinear multivariable systems under input-output feedback linearization is to allow plant uncertainty to be propagated through the control design, yielding an uncertainty description of the closed-loop in polytopic form, as presented by Botto et al. (2001).

Recently, Taghieh and Shafiei (2021a) proposed an observer-based robust model predictive control scheme to control a class of switched nonlinear systems in the presence of time delay and parametric uncertainties under arbitrary switching in addition to proposing a static output feedback controller (Taghieh and Shafiei, 2021b) for a class of switched nonlinear systems subject to time-varying delay and uncertainties under asynchronous switching. Zhang et al. (2022) addressed nonlinear systems with mismatched uncertainties under input/output quantization proposing adaptive output feedback control. Fractional parametric uncertainties and distributed delays in nonlinear systems together with time delay, parametric uncertainties and actuator faults were just addressed by Sweetha et al. using a non-fragile fault-tolerant controller, which makes the system asymptotically stable with the specified mixed H∞ and passive performance index (Sweetha et al., 2022). Wei et al. (2022) sought to control uncertain nonlinear processes using neural networks incorporating into the control loop an adaptive neural network embedded contraction-based controller (to ensure convergence to time-varying references) and an online parameter identification module coupled with reference generation (to ensure modeled parameters converge those of the physical system). Wang et al. (2009) investigated quasi-Hamiltonian systems with parametric uncertainty using the stochastic averaging method and stochastic dynamical programming principle. A particular strength of the work lies in two examples given to illustrate the proposed control procedure and its robustness. Mahmoodabadi and Andalib Sahnehsaraei (2021) introduced a new online optimal control based on the input–output feedback linearization and a multi-crossover genetic algorithm for under-actuated nonlinear systems having parametric uncertainties. Optimal control problems with bounded uncertainties on parameters were addressed using interval arithmetics by Etienne et al. where an interval method based on Pontryagin’s Minimum Principle (as proposed here) is proposed in Bertin et al. (2021) to enclose the solutions of an optimal control problem with embedded bounded uncertainties. This method is used to compute an enclosure of all optimal trajectories of the problem and open-loop and closed-loop enclosures meant to validate an optimal guidance algorithm on a concrete system with inaccurate knowledge of the parameters.

Next-generation methods are required that apply mathematically optimal results yet retain the simplicity of analytics solutions obfuscating numerical (or otherwise more complicated) methods and providing further advancements in autonomous navigation. The current movement toward the utilization of very small vehicles is accompanied by very limited computational resources while maintaining autonomy, robustness, and accuracy. Newly proposed methods and algorithms for autonomous guidance and control are presented in direct, critical comparison to the recent research trends of both academia and industry, presuming utilization of noisy sensors, for example, star trackers, rate gyroscopes, inertial measurement units, and global navigation systems, amongst other sensors in multi-sensor-based architectures for vehicle navigation. Intelligence methods permitting systems to learn real-time optimal solutions (analytically) are preferred.

Proposed novelties:

1. A brief methodological recitation of five disparate incarnations of optimal control and their direct comparison to classical feedback control as a benchmark (the P + V proportional plus velocity controller): 1) control-minimizing open-loop optimal, 2) linear-quadratic optimal regulator, 3) time-optimal, 4) real-time optimal, and 5) real-time optimal with singular switching. Methods 3, 4, and 5 involve no feedback control gains tuning.
2. Direct comparison of the efficacy of each of the five methods listed in item #1 controlling *linear* double-integrator plants, where comparison is made using state accuracy, rate accuracy, control (fuel) usage, and computational runtime (as a manifestation of computational burden).
3. Direct comparison of the efficacy of each of the five methods listed in item #1 controlling double-integrator plants, *including nonlinear transport theorem cross-products of motion* induced by measurement in rotating reference frames, where comparison is made using state accuracy, rate accuracy, control (fuel) usage, and computational runtime (as a manifestation of computational burden). In item #3, the *linear control designs are used on the nonlinear plants* to evaluate the error resulting from using linear control designs in the real-world on nonlinear systems.
4. Direct comparison of the efficacy of each of the five methods listed in item #1 controlling double-integrator plants, *including nonlinear transport theorem cross-products of motion* induced by measurement in rotating reference frames, where comparison is made using state accuracy, rate accuracy, control (fuel) usage, and computational runtime (as a manifestation of computational burden). *Unlike item #3 above, nonlinear decoupling control stemming from the solution to the minimum-control optimization problem is introduced* to each control methodology by utilizing the optimal rate trajectories that result from the original open-loop optimization problem that minimizes control effort. This nonlinear control utilizing the constrained optimization problem results (linear control and nonlinear combinations of the optimal trajectories) may be considered the largest contribution to the article.
5. Items #3 and #4 are both repeated to evaluate the deleterious effects on each method of *noisy sensors and random uniformly varying system mass and mass moments of inertia.*

Motivated to develop simple methods that flow from the solution of constrained optimization problems yet do not necessitate numerical solutions leads to arguably, the most interesting proposal: Utilization of analytic solutions to the constrained optimization problem in either a feedforward or feedback sense applied to full nonlinear, coupled guidance and control problems, specifically including the transport theorem coupling cross-products for rotation and translation, respectively. This method is mathematically developed in section 2, resulting in proposals for both feedforward and feedback methods.

**FIGURE 2:**
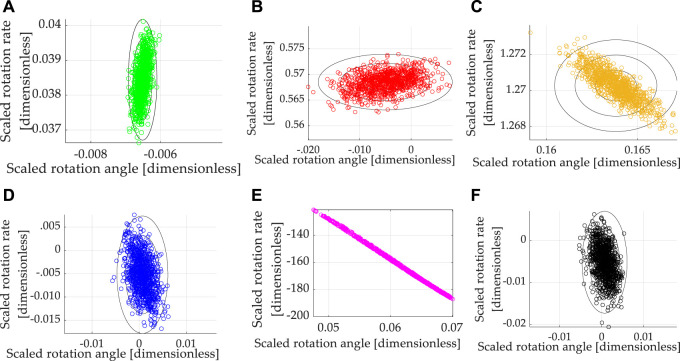
Scatter plots displaying the results of 1,000 simulation runs (per case) with randomly (uniformly) varied mass and mass moments ±10% . **(A)** classical P + V, **(B)** LQR optimal PD, **(C)** time-optimal control, **(D)** open-loop optimal, **(E)** real-time optimal (RTOC), and **(F)** switched RTOC.

Section 2 includes brief derivations of each respective approach as briefly as practicable, while section 3 provides the results of implementing each disparate methodology. Tables of variable definitions and nomenclature have been placed throughout the manuscript: Tables 1-6, while Table 5 articulates necessary methods for repeating the presented work. Table 7 summarizes Monte Carlos analysis of parameter variations from Figure 2, while Table 8 summarizes percent performance improvements for each of the six evaluated techniques.

**TABLE 1:** Double-integrator plant ten-run mini-Monte Carlo analysis (faults occurred after first simulation run) executed in MATLAB^®^/SIMULINK^®^ R2021b (9.11.0.1769968) whose machine precision $eps=2.2204\times 10^{-16}$ .

Method^a^	State error	Rate error	Cost	Runtime
${\left[T\right]}^{-1}$	Fault	Fault	Fault	Fault
1\[T]	0.000052	−0.0048598	4.0281	1.6221
inv[T]	Fault	Fault	Fault	Fault
pinv[T]	0.001169	−0.003941	4.0281	1.5589
LU Inverse [T]	Fault	Fault	Fault	Fault

a Real-time optimal control (with singular switching).

**TABLE 2:** Proximal variable definitions.

Variable	Definition	Variable	Definition
${\bar{x}}_{d}$	Desired state trajectory	$\bar{\bar{\xi }}$	Critical damping ratio
${\bar{\bar{K}}}_{P}$	Proportional gain	${\bar{\omega }}_{n}$	Natural frequency
${\bar{\bar{K}}}_{v}$	Velocity gain	$t_{s}$	Settling time

Such tables are distributed throughout the manuscript to increase the ease of reading, while a combined master table of definitions is included in the Supplementary Appendix.

**TABLE 3:** Proximal variable definitions.

Variable	Definition	Variable	Definition
$\bar{\bar{A}}$	State transition matrix	J	Cost function
$\bar{\bar{B}}$	Control matrix	$t_{f}$	Final time
$\bar{\bar{K}}$	Gain matrix	∞	Infinity
$\bar{\bar{Q}}$	State weighting matrix	sgn	Signum function
$\bar{\bar{R}}$	Control weighting matrix	p (t)	Parameters (co-states)
$\bar{\bar{P}}$	Covariance matrix	$b_{i}$	Control coefficients

**TABLE 4:** Proximal variable definitions.

Variable	Definition	Variable	Definition
J	Cost function	${\bar{u}}^{*}$	Optimal control
dt	Differential time	${\dot{\bar{v}}}^{*}$	Optimal (angular) acceleration
t	Time	${\bar{v}}^{*}$	Optimal (angular) velocity
$\bar{u}$	Control	${\bar{x}}^{*}$	Optimal (angular) position
$\bar{x}\left(t\right)$	Current position	$\bar{a}$ , $\bar{b}$ , $\bar{c}$ , $\bar{d}$	Integration constants
$\bar{v}\left(t\right)$	Current velocity	$\hat{a}$ , $\hat{b}$ , $\hat{c}$ , $\hat{d}$	Integration constant estimates

**TABLE 5:** Proximal variable definitions.

Variable	Definition	Variable	Definition
$\bar{F}$	Externally applied forces	$\bar{T}$	Externally applied torques
$\bar{\bar{m}}$	Mass	$\bar{\bar{J}}$	Mass moment of inertia
$\bar{a}=\ddot{\bar{x}}$	Translational acceleration	$\bar{\alpha }=\ddot{\bar{\theta }}$	Rotational acceleration
$\frac{d^{2}\bar{x}}{dt^{2}}=\dot{v}$	Translational acceleration	$\frac{d^{2}\bar{\theta }}{dt^{2}}=\dot{\bar{\omega }}$	Rotational acceleration
$\bar{r}$	Radius vector relative to rotating frame	$\bar{\omega }=\dot{\bar{\theta }}$	Rotational velocity
$\bar{v}$	Velocity vector relative to rotating frame	$\bar{\theta }$	Displacement angle

Such tables are distributed throughout the manuscript to increase the ease of reading, while a combined master table of definitions is included in the Supplementary Appendix.

**TABLE 6:** Proximal variable definitions.

*Variable*	*Definition*	*Variable*	*Definition*
${\left[T\right]}^{-1}$	*MATLAB inverse*	*m*	*Mass*
1\[T]	*MATLAB inverse*	*J*	*Mass moments*
inv[T]	*MATLAB inverse*	ω	*Angular velocity*
pinv[T]	*MATLAB pseudo-inverse*	r	*Position vector*
*lu(* [T])	*MATLAB LU-inverse*	v	*Translational velocity*
$u_{transport}$	*Feedback decoupler*	${\text{ω}}^{\text{*}}$	*Optimal angular velocity*
$u_{transport}^{*}$	*Feedforward (decoupler)*	${\text{r}}^{\text{*}}$	*Optimal position vector*
		${\text{v}}^{\text{*}}$	*Optimal Translational velocity*
*u*	*Control, the sum of* *Eq. 18* *or* *Eq. 19* *with* *Eqs 5, 8, 12, 14* *, or* *Eq. 15*		

**TABLE 7:** One-thousand-run (respectively) Monte Carlo analysis.

Method	State error	Rate error	Cost	Runtime
Classical *p* + V	−0.0065157	0.038442	25.3181	1.2342
LQR Optimal PD	−0.0050504	0.56851	75.5278	1.23
Time-optimal control	0.16381	1.2703	1.3643	1.3055
Open loop optimal^a^	0.00069197	−0.0052251	4.0281	1.2335
Real-time optimal (RTOC)^a^	0.062551	−165.1258	40,959.5421	1.2818
Switched RTOC^a^	0.00066117	−0.0051746	4.0281	1.1068

a Real-time optimal control (with and without switching) and open-loop optimal control are visually indistinguishable from one another in the graphic depiction.

**TABLE 8:** Percentage change in performance in one-thousand-run (respectively) Monte Carlo analysis: double-integrator plant (*with* transport theorem) with control design based off double-integrator *with* transport theorem and noisy, mixed sensors (state and rate).

Method	State error	Rate error	Cost	Runtime
Classical *p* + V	—	—	—	—
LQR Optimal PD	−22%	1,379%	198%	0%
Time-optimal control	2,614%	3,204%	−95%	6%
Open loop optimal ^a^	−111%	−114%	−84%	0%
Real-time optimal (RTOC) ^a^	1,060%	429,645%	161,680%	4%
Switched RTOC ^a^	−111%	−114%	−84%	−10%

a Real-time optimal control ${\text{u}}_{\text{total}}^{\text{*}}=\hat{a}t+\hat{b}+{\bar{\omega }}^{*}\times \bar{\bar{J}}{\bar{\omega }}^{*}$ (with and without switching) and open-loop optimal control are visually indistinguishable from one another in the graphic depiction.

## 2 Materials and Methods

Motion (both translational and rotational) is governed by so-called double-integrator dynamics where the integral of the applied forces vector (inversely scaled by the mass or mass moments, respectively) is the velocity vector and the integral of the velocity vector (translational or rotational) is the displacement vector. Each vector is relative to an inertial (non-rotating) reference frame, while the expression of the vectors in the coordinates of the basis vectors of rotating reference frames necessitates the inclusion of the transport theorem, which articulates the induced motion of the rotating reference frame in cross-products that make the results nonlinear and coupled. The three degrees of rotational motion are coupled to each other by the transport theorem, and the three degrees of translational motion are coupled to each other as well. Furthermore, the three degrees of translation are coupled nonlinearly to the three degrees of rotation, particularly through the angular velocity vector. Especially since this nonlinear coupling is a foremost challenge that is often deemed insurmountable by analytic methods, the foremost subsections of this part of the manuscript begin so. The Materials and Methods section of the manuscript is described with sufficient details to allow readers to replicate and build on the published results.

### 2.1 Double-Integrator Based Plant Equations

Eq. 1 illustrates the fundamental relationships of both translational and rotational motion may be expressed as so-called double-integrators, meant to mean the twice integration of the applied force or torques produces the respective translational or rotational displacement.

$$\bar{F}=\bar{\bar{m}}\bar{a}=\bar{m}\ddot{\bar{x}}=\bar{m}\frac{d^{2}\bar{x}}{dt^{2}}\;\;↔\;\;\bar{T}=\bar{\bar{J}}\bar{\alpha }=\bar{\bar{J}}\dot{\bar{\omega }}=\bar{\bar{J}}\ddot{\bar{\theta }}=\bar{\bar{J}}\frac{d^{2}\bar{\theta }}{dt^{2}},$$ (1)

Eq. 1 comprises two sets of three equations each for translation and rotation combining for six equations of mechanical motion. For simplicity of expression, states referred to generically as *x* can represent rotations ( θ ) with regards to the basic, shared motion described by the double-integrator. The transport theorem described next will generate differences in the governing equations for translation and rotation.

### 2.2 Transport Theorem Cross-Product Coupled Motion Expressed in Rotating Reference Frames

Attach three mutually perpendicular unit vectors to each frame: the non-rotating inertial frame and body-fixed frame. The meaning of differentiation of vectors when specification is made of differentiation with respect to a specific frame. Both rotational and translational motion relative to the non-rotating reference frame may be represented by double-integrators in accordance with Eq. 2.

$$\bar{\bar{m}}{\bar{a}|}_{relative}=\bar{\bar{m}}{\ddot{x}|}_{relative}=\bar{\bar{m}}{\frac{d^{2}x}{dt^{2}}|}_{relative},$$ (2)

Theorem 1. *Transport Theorem. The derivative of any vector expressed in the coordinates of a rotating reference frame equals the sum of the derivative relative to a non-rotating reference frame plus the cross-product of the angular velocity and the vector.*

Proof of Theorem 1. The proof of this well-known theorem is provided by Kinematics Handout - MIT OpenCourseWare, 2021. The tedious process may be summarized as follows: 1) express the position vector with respect to the non-rotating inertial reference frame; 2) differentiate to find the expression for velocity remembering to differentiate both the component measurements and the unit vectors; 3) simplify and substitute the defined unit vectors, define the angular velocity in the direction perpendicular to the two-dimensional space of rotation; and 4) substitute the newly defined angular velocity to arrive at the transport theorem as expressed in Eq. 3. □

$$\bar{\bar{J}}\frac{d\bar{\omega }}{dt}=\bar{\bar{J}}{\frac{d\bar{\omega }}{dt}|}_{relative}+\bar{\omega }\times J\bar{\omega },$$ (3)

The inclusion of theorem 1, despite being very well-known, is purposely performed to emphasize the most novel proposals presented. In particular, Eqs. 2 and 3 are ubiquitously approximated first by Eq. 1 and also often by linearization of Taylor’s Series of each equation, respectively.

#### 2.2.1 Euler’s Moment Equations of Rotation Expressed in a Rotating Reference Frame

Externally applied torques, $\bar{T}$ change angular momentum $\bar{\bar{J}}\frac{d\bar{\omega }}{d\text{t}}$ permitting the substitution into Eq. 3, resulting in Euler’s nonlinear moment equation in Eq. 4. A system is called linear if it has two mathematical properties: homogeneity and additivity in accordance with the principle of superposition. If two or more solutions to an equation or set of equations can be added together so that their sum is also a solution, linearity may be asserted. In other words, two or more states of the system must be added together to create an additional state. Adding two single-channel angles cannot also be a solution without accounting for the presence of the other two channels’ states.

An easy way to understand the nonlinear nature of each motion channel induced by the linear cross-product transformation is to recall the mathematical definition: a×b=ab|sinθ| , while f (x + y) = f(x) + f(y) is a simple counterexample showing that this function f is not linear: $\sin\left(\theta_{1}+\theta_{2}\right)\neq \sin\left(\theta_{1}\right)+\sin\left(\theta_{2}\right)$ . An often-confused notion is the fact that the cross-product is a linear transformation, but nonetheless, each motion channel is evidently nonlinear in the states (evidenced by the presence of multiplicative state pairs and cross-coupled states in each channel. From the perspective of linear algebra, the matrix representation of the cross-product is skew-symmetric and has determinant zero, so it will not always have a solution.

$$\begin{array}{c} \bar{T}=\bar{\bar{J}}\dot{\bar{\omega }}+\bar{\omega }\times \bar{\bar{J}}\bar{\omega }\\ \left[\begin{array}{c} \tau_{x}\\ \tau_{y}\\ \tau_{z} \end{array}\right]=\left[\begin{array}{c} J_{xx}{\dot{\omega }}_{x}+J_{xy}{\dot{\omega }}_{y}+J_{xz}{\dot{\omega }}_{z}-J_{xy}\omega_{x}\omega_{z}-J_{yy}\omega_{y}\omega_{z}-J_{yz}\omega_{z}^{2}+J_{xz}\omega_{x}\omega_{y}+J_{zz}\omega_{z}\omega_{y}+J_{yz}\omega_{y}^{2}\\ J_{yx}{\dot{\omega }}_{x}+J_{yy}{\dot{\omega }}_{y}+J_{yz}{\dot{\omega }}_{z}-J_{yz}\omega_{x}\omega_{y}-J_{zz}\omega_{x}\omega_{z}-J_{xz}\omega_{x}^{2}+J_{xx}\omega_{x}\omega_{z}+J_{xy}\omega_{z}\omega_{y}+J_{xz}\omega_{z}^{2}\\ J_{zx}{\dot{\omega }}_{x}+J_{zy}{\dot{\omega }}_{y}+J_{zz}{\dot{\omega }}_{z}-J_{xx}\omega_{x}\omega_{y}-J_{xz}\omega_{y}\omega_{z}-J_{xy}\omega_{y}^{2}+J_{yy}\omega_{x}\omega_{y}+J_{yz}\omega_{z}\omega_{x}+J_{xy}\omega_{x}^{2} \end{array}\right] \end{array},$$ (4)

It should be noted the dominant double-integrator dynamics are embodied in $J\dot{\omega }$ in Eq. 4, while the additional accelerations due to the transport theorem are embodied in the coupling cross-product term $\bar{\omega }\times \bar{\bar{J}}\bar{\omega }$ . Control designs based on the double-integrator dynamics alone are hypothesized to have less efficacy than proposed techniques that utilize optimization and the transport theorem terms.

#### 2.2.2 Newton’s Equations of Translation Expressed in a Rotating Reference Frame

Performing similar expression of translational motion in non-rotating inertial frames as just performed in section 2.2.1 for rotational motion leads to Eq. 5 for Newton’s equations of translational motion.

$$\bar{F}=\underset{Relative}{\underset{︸}{\bar{\bar{m}}\bar{a}}}+\underset{Euler}{\underset{︸}{\bar{\bar{m}}\frac{d\bar{\omega }}{dt}}}\times \bar{r}+\underset{Coriolis}{\underset{︸}{2\bar{\bar{m}}\bar{\omega }\times \bar{v}}}+\underset{Centrifigual}{\underset{︸}{\bar{\bar{m}}\bar{\omega }\times \bar{\omega }\times \bar{r}}},$$ (5)

Notice the dominant double-integrator dynamics relative to the rotating reference frame in Eq. 5 are embodied in ma , while the additional accelerations due to the transport theorem are embodied in the coupling cross-product terms: Euler ( $\bar{\bar{m}}\frac{d\bar{\omega }}{dt}\times \bar{r}$ ), Coriolis ( $2\bar{\bar{m}}\bar{\omega }\times \bar{v}$ ), and centrifugal ( $\bar{\bar{m}}\bar{\omega }\times \bar{\omega }\times \bar{r}$ ). Control designs based on the double-integrator dynamics alone are hypothesized to have less efficacy than proposed techniques that utilize optimization and the transport theorem terms.

#### 2.2.3 Impacts on Control Design

Neglecting the cross-products of acceleration resulting from the transport theorem reduces both Eqs. 4 and 5 to the double-integrators of Eqs. 1 and 2. The goal of this research is to develop controls (for applied forces $\bar{F}$ and applied torques $\bar{T}$ ) that account for the nonlinear, coupling cross-products produced by the application of the transport theorem. Typically, nonlinearities like those presented in Eqs. 4 and 5 caused by transport theorem are simplified by assumption, neglected altogether, or linearized to permit linear control design. Subsequently, the linear controllers are applied to the nonlinear systems and augmented as necessary to improve performance. Instead, the optimal trajectories that result from the solution of constrained optimization problems (for translation and rotation, respectively) are combined to form new nonlinear controls. The exact form of the nonlinear equation is used to form the new nonlinear control components where the motion states are taken from the solution to the constrained optimization problem.

### 2.3 Classical Position Plus Velocity (P + V) Feedback Control

Proportional plus velocity control (Chai et al., 2019) utilizes proportional control by forming a state error scaled by a proportional gain adding a negative gained value of velocity (translational or rotational), as elaborated in Eq. 5. The velocity channel is not a differentiated version of the position or angle channel, as is the case with classical cascaded control topologies of PD, PI, and PID types (proportional plus derivative, proportional plus integral, and proportional plus integral plus derivative, respectively).

$$\begin{array}{c} \bar{\bar{J}}\ddot{\bar{x}}+{\bar{\bar{K}}}_{V}\dot{\bar{x}}+{\bar{\bar{K}}}_{P}\bar{x}={\bar{\bar{K}}}_{P}{\bar{x}}_{d}\;\;↔\;\;\bar{u}={\bar{\bar{K}}}_{p}\left({\bar{x}}_{d}-\bar{x}\right)-{\bar{\bar{K}}}_{v}\dot{\bar{x}}\\ \frac{\bar{x}\left(s\right)}{{\bar{x}}_{d}\left(s\right)}=\frac{{\bar{\bar{K}}}_{p}}{\bar{\bar{J}}s^{2}+{\bar{\bar{K}}}_{v}s+{\bar{\bar{K}}}_{p}}\to C.E.:\;{s^{2}+{\bar{\bar{K}}}_{v}s+{\bar{\bar{K}}}_{p}|}_{I=1}=s^{2}+2\xi \omega_{n}s+\omega_{n}^{2} \end{array}.$$ (6)

Gains were tuned for performance specification by equating the ubiquitous closed-loop system Eq. 5 to the performance specified, where *C.E.* annotates the characteristic equation. The desired rise time established the system natural frequency per $t_{r}=\frac{1.8}{{\bar{\omega }}_{n}}$ , where ${\bar{\omega }}_{n}\approx {\bar{\omega }}_{b}$ is the desired control bandwidth; therefore, ${\bar{\omega }}_{n}=\frac{1.8}{t_{r}}\to {\bar{\bar{K}}}_{p}={\bar{\omega }}_{n}^{2}$ . Settling time: oscillation stabilize within 2–5% percent of steady state $t_{s}=\frac{4.6}{\bar{\bar{\xi }}{\bar{\omega }}_{n}}$ $\to \bar{\bar{\xi }}=\frac{4.6}{t_{s}{\bar{\omega }}_{n}}\to {\bar{\bar{K}}}_{v}=2\bar{\bar{\xi }}{\bar{\omega }}_{n}$ .

Elimination of differentiation in the derivative channel often bestows relative advantage in tracking desired velocity trajectories. Another approach is the optimize gain selection, and this alternative approach is called the linear quadratic regulator.

### 2.4 Linear-Quadratic Optimal Regulator of Proportional Derivative Type (Murray, 2010)

Eqs. 3 and 4, representing the full, nonlinear, coupled equations of motion in six degrees, may be linearized and be expressed in the form displayed in Eq. 6. This linearization is the basis for the word “linear” in the LQR title. The word “quadratic” refers to selecting gains *K* that minimize a quadratic cost function displayed in Eq. 7. The LQR solution (Kwakernaak and Sivan, 1972; NASA, 2021a) only bestows optimal solutions for control gains of the form Eq. 8 that minimizes the quadratic cost simultaneously satisfying the (linearized) dynamic constraints displayed in Eq. 5.

$$\dot{\bar{x}}=\bar{\bar{A}}\bar{x}+\bar{\bar{B}}\bar{u},$$ (7)

$$J=\overset{\infty }{\underset{0}{\int }}\left({\bar{x}}^{T}\bar{\bar{Q}}\bar{x}+{\bar{u}}^{T}\bar{\bar{R}}\bar{u}\right)dt,$$ (8)

$$\bar{u}=-\bar{\bar{K}}\bar{x}.$$ (9)

The control designer may select the state weighting matrix $\bar{\bar{Q}}$ and the control weighting matrix $\bar{\bar{R}}$ to penalize the state errors and the control effort, respectively. In section 3, equally weighted identity matrices were chosen for both $\bar{\bar{Q}}$ and $\bar{\bar{R}}$ . This choice facilitates a multi-faceted comparison in section 3 that does not solely focus on tracking errors or costs. The gains $\bar{\bar{K}}$ are found using Eq. 9, where the matrix $\bar{\bar{P}}$ is first found by solving the algebraic relation in Eq. 10, often referred to as a Riccati equation which is most often solved iteratively by a computer (the MATLAB^®^/SIMULINK^®^ *lqr* command).

$$\bar{\bar{K}}={\bar{\bar{R}}}^{-1}\left({\bar{\bar{B}}}^{T}\bar{\bar{P}}\right)$$ (10)

$${\bar{\bar{A}}}^{T}\bar{\bar{P}}+\bar{\bar{P}}\bar{\bar{A}}-\bar{\bar{P}}\bar{\bar{B}}{\bar{\bar{R}}}^{-1}{\bar{\bar{B}}}^{T}\bar{\bar{P}}+\bar{\bar{Q}}=\bar{0}$$ (11)

### 2.5 Time-Optimal Control (Murray, 2010)

Minimizing a non-quadratic cost function comprised of only the final time (as displayed in Eqs. 11 and 12) constrained with the linearized dynamics of Eq. 6 with costate parameters *p(t)* used in the Hamiltonian problem formulation leads to time-optimal control (Flugge-Lotz, 1953; Pontryagin et al., 1962; Boltyanskii, 1971; Sands et al., 2009; Sands and Ghadawala, 2011; Duprez et al., 2017; Heidlauf and Cooper, 2017; Baker et al., 2018; Sands, 2019; Smeresky et al., 2020; Arguchintsev and Poplevko, 2021; Malecek, 2021; Srochko et al., 2021).

$$J=\overset{\infty }{\underset{0}{\int }}t_{f}dt,$$ (12)

$$\bar{u}=sgn\left(<p\left(t\right),b_{i}>\right)=\{\begin{array}{c} 1\\ -1\\ 0 \end{array}\;\;\;\;\;\;\begin{array}{c} if\\ if\\ if \end{array}\begin{array}{c} <p\left(t\right),b_{i}>>0\\ <p\left(t\right),b_{i}><0\\ <p\left(t\right),b_{i}>=0 \end{array}.$$ (13)

Simulation subsystems depicted in the appendix execute a *bang-bang control* where maximal application of control is normalized to unity such that desired unity state and unity time is achieved to aid comparison to the other optimization approaches. One key feature of *bang-bang* control is the neglecting of the rate end condition leading to a so-called *bang-off-bang* control, which is not treated here.

### 2.6 Open Loop Minimum-Control Optimization (Pontryagin et al., 1962; Ross, 2015)

Minimizing only the control effort alone (not the state errors) (Sands, 2019) in accordance with Eq. 13 constrained by the double-integrator dynamics of Eq. 2 for specified initial and final conditions permits the solution of a two-point boundary value problem producing optimal control, acceleration, rate, and state profiles displayed in Eq. 14, respectively. Normalization for unity masses or mass moments is included, thus, control and acceleration equations are identical, where non-normalized control may be expressed by scaling the control equation by the masses or mass moments, respectively.

By specifying quiescent initial conditions and using variable scaling and balancing to normalize the final position coordinate to unity, the constants in Eq. 14 may be solved, resulting in Eq. 15, where *a =* −*12, b = 6*, and *c = d = 0*. It should be noted that states are not penalized in the cost function, instead only solution forms that satisfy the boundary values are produced by the two-point boundary value problem from the initial point (*x(0),v(0)*) *=* (*0,0*) to the final point (*x(1), v(1)*) *=* (*1,0*), thus, there is no need to solve an algebraic Riccati equation to produce the optimal control, where an additional benefit of this optimization approach includes the production of optimal state trajectories that will prove useful to decouple the nonlinear coupling effects of the transport theorem described in section 2.2. Scaling and balancing must be performed to normalize the initial and final conditions to zero and unity, and the operations are explained in section 2.11. The solution to the constrained optimization problem listed in Eqs. 2 and 13 was solved analytically and presented recently by Sands (2021) for virtual sensoring, and that solution is presented here in Eqs. 14 and 15. The mathematical development is intentional since 1) the development is well-articulated by Sands (2021) and 2) increased focus on the utilization of these results toward nonlinear equations of motion (presented in Section 2.9).

$$J=\frac{1}{2}\overset{\infty }{\underset{0}{\int }}\left({\bar{u}}^{T}\bar{u}\right)dt,$$ (14)

$${\bar{u}}^{*}=\bar{a}t+\bar{b},{\dot{\bar{v}}}^{*}=\bar{a}t+\bar{b},{\bar{v}}^{*}=\frac{1}{2}\bar{a}t^{2}+\bar{b}t+\bar{c},{\bar{x}}^{*}=\frac{1}{6}\bar{a}t^{3}+\frac{1}{2}\bar{b}t^{2}+\bar{c}t+\bar{d},$$ (15)

$${\bar{u}}^{*}=-\bar{12}t+\bar{6},{\dot{v}}^{*}=-\bar{12}t+\bar{6},{\bar{v}}^{*}=-\bar{6}t^{2}+\bar{6}t,{\bar{x}}^{*}=-\bar{2}t^{3}+\bar{3}t^{2}.$$ (16)

The open-loop optimal solution embodied in Eqs. 14 and 15 may be updated in real-time using state feedback resulting in real-time optimal control presented in Section 2.7. These optimal states in Eq. 15 are used to form nonlinear controls in Section 2.9.

### 2.7 Real-Time Optimal Control

A corollary is to the open-loop minimum-control optimization in section 2.6 augments the approach with feedback while maintaining the remaining portions of the problem approach. The solution for the constants in between Eqs. 14 and 15 may be accomplished in real-time using feedback but *asserting the current position and velocities (translational and rotational) are the initialization points of a new two-point boundary value problem*. Eq. (16) may be written in a matrix-vector form as Eq. 17, permitting real-time solution for the integration constants in the vector by inverting the matrix and pre-multiplying both sides of the equation as depicted in (17). Notice the form of the control derived in Eq. 17 is the same as Eq. 14 in section 2.6, where the constants in the optimal solutions are solved in real-time.

$$v^{*}=\frac{1}{2}at^{2}+bt+c,x^{*}=\frac{1}{6}at^{3}+\frac{1}{2}bt^{2}\&v^{*}\left(t_{f}\right)=\frac{1}{2}a+b=0,x^{*}\left(t_{f}\right)=\frac{1}{6}a+\frac{1}{2}b=1,$$ (17)

$$\underset{\left[T\right]}{\underset{︸}{\left[\begin{array}{cccc} \frac{t_{0}^{2}}{6} & \frac{t_{0}^{2}}{2} & t_{0} & 1\\ \frac{t_{0}^{2}}{2} & t_{0} & 1 & 0\\ \frac{1}{6} & \frac{1}{2} & 1 & 1\\ \frac{1}{2} & 1 & 1 & 0 \end{array}\right]}}\underset{\left\{p\right\}}{\underset{︸}{\left\{\begin{array}{c} a\\ b\\ c\\ d \end{array}\right\}}}=\underset{\left\{b\right\}}{\underset{︸}{\left\{\begin{array}{c} {\bar{x}}_{0}\\ {\bar{v}}_{0}\\ 1\\ 0 \end{array}\right\}}}\to \left\{\begin{array}{c} \hat{a}\\ \hat{b}\\ \hat{c}\\ \hat{d} \end{array}\right\}={\left[\begin{array}{cccc} \frac{t_{0}^{2}}{6} & \frac{t_{0}^{2}}{2} & t_{0} & 1\\ \frac{t_{0}^{2}}{2} & t_{0} & 1 & 0\\ \frac{1}{6} & \frac{1}{2} & 1 & 1\\ \frac{1}{2} & 1 & 1 & 0 \end{array}\right]}^{-1}\left\{\begin{array}{c} \bar{x}\left(t\right)\\ \bar{v}\left(t\right)\\ 1\\ 0 \end{array}\right\}\text{ and }{\bar{u}}^{*}\equiv \hat{a}t+\hat{b}.$$ (18)

One key feature of the open-loop solution method using a two-point boundary-value problem is the enforcement of end conditions producing optimal trajectories for state ( $\bar{x}$ *), rate ( $\bar{v}$ *), acceleration ( ${\dot{\bar{v}}}^{*}$ ), and jerk ( ${\ddot{\bar{v}}}^{*}$ ), in addition to the formulation of an optimal control, u*. These signals yield the opportunity to formulate decoupling control components to mitigate the transport theorem (illustrated in section 2.9).

### 2.8 Real-Time Optimal Control With Singular Switching

Highlighting the matrix inverse in Eq. 17, the possibility of issues inverting a poorly conditioned or rank-deficient matrix may be addressed by monitoring matrix conditioning or determinant and switching away from the feedback solution when encountering rank-deficient instances in favor of the optimal solution in Eq. 15.

#### 2.8.1 Matrix Inverse Formulas

Five disparate methods to invert the [T] matrix were investigated, as listed in Eq. 18. Matrix inversion methods already coded in MATLAB/SIMULINK: ${\left[T\right]}^{-1}$ , 1\[T] , inv[T] , pinv[T] , LU Inverse [T] . Each method has specific strengths and weaknesses expressed in state error, rate error, control effort (quadratic cost), and runtime, as displayed in Table 1. In several instances, the simulation would fault as a result of encountering matrix singularity.

### 2.9 Nonlinear Transport Theorem Decoupling (Recall Transport Theorem in Section 2.2)

As mentioned in Section 2.6, the desire is to use the results of Sections 2.7 , 2.8 applied to nonlinear dynamics coupled by transport theorem. Section 2.2 describes the nonlinear coupling effects of measuring motion in coordinates of rotating reference frames extracted for highlighting in Eq. 18 for translation and rotation, respectively. These effects were neglected when optimizing the double-integrator–based systems of equations or simplified by linearization in other instantiations. Taking advantage of the results in Sections 2.6, 2.7, nonlinear decoupling control components may be formulated using the optimal trajectories as displayed in Eq. 18, *where each component (translation and rotation, respectively) should be added to augment the control in* *Eq. 17* *.*

$$\begin{array}{c} \begin{array}{c} Rotation:\;u_{nonlinear}=\;\bar{\omega }\times \bar{\bar{J}}\bar{\omega }\\ Translation:u_{nonlinear}=\bar{\bar{m}}\frac{d\bar{\omega }}{dt}\times \bar{r}+2\bar{\bar{m}}\bar{\omega }\times \bar{v}+\bar{\bar{m}}\bar{\omega }\times \bar{\omega }\times \bar{r} \end{array}\}\;Feedback\\ \begin{array}{c} Rotation:\;u_{nonlinear}={\bar{\omega }}^{*}\times \bar{\bar{J}}{\bar{\omega }}^{*}\\ Translation:\;u_{nonlinear}=\bar{\bar{m}}\frac{d{\bar{\omega }}^{*}}{dt}\times {\bar{r}}^{*}+2\bar{\bar{m}}{\bar{\omega }}^{*}\times {\bar{v}}^{*}+\bar{\bar{m}}{\bar{\omega }}^{*}\times {\bar{\omega }}^{*}\times {\bar{r}}^{*} \end{array}\}\;Feedforward \end{array},$$ (19)

It is proposed to take the nonlinear control components in Eq. 18 formulated using the optimal *trajectories* from Eq. 18 and augment them with Eq. 17’s optimal *control* solutions to comprise the *total control* for rotation and translation, respectively, displayed in Eq. 19.

$$\begin{array}{c} \begin{array}{c} Rotation:\;u_{total}^{*}=\;\hat{a}t+\hat{b}+\bar{\omega }\times \bar{\bar{J}}\bar{\omega }\\ Translation:u_{total}^{*}=\hat{a}t+\hat{b}+\bar{\bar{m}}\frac{d\bar{\omega }}{dt}\times \bar{r}+2\bar{\bar{m}}\bar{\omega }\times \bar{v}+\bar{\bar{m}}\bar{\omega }\times \bar{\omega }\times \bar{r} \end{array}\}\;\;Feedback\\ \begin{array}{c} Rotation:\;u_{total}^{*}=\hat{a}t+\hat{b}+{\bar{\omega }}^{*}\times \bar{\bar{J}}{\bar{\omega }}^{*}\\ Translation:\;u_{total}^{*}=\hat{a}t+\hat{b}+\bar{\bar{m}}\frac{d{\bar{\omega }}^{*}}{dt}\times {\bar{r}}^{*}+2\bar{\bar{m}}{\bar{\omega }}^{*}\times {\bar{v}}^{*}+\bar{\bar{m}}{\bar{\omega }}^{*}\times {\bar{\omega }}^{*}\times {\bar{r}}^{*} \end{array}\}\;Feedforward \end{array},$$ (20)

The distinction between feedforward and feedback is determined by the chosen manner of decoupling the nonlinear transport theorem. One option is feedback decoupling, where feedback states are combined (for example, for rotation) as $\bar{\omega }\times \bar{\bar{J}}\bar{\omega }$ and added to the optimal control to form a new nonlinear control. On the other hand, feedforward decoupling (used in section 3) combines the optimal equations of state from Eqs. 15 and 16, respectively, combined as ${\bar{\omega }}^{*}\times \bar{\bar{J}}{\bar{\omega }}^{*}$ and augments the optimal controls with this new nonlinear control. The efficacy of this latter suggestion is evaluated in section 3.

Section 3 validates the proposed developments culminating in the combination of real-time optimal control (Eq. 17) together with transport theorem decoupling in Eq. 19, specifically for rotation using feedforward: ${\text{u}}_{\text{total}}^{\text{*}}=\hat{a}t+\hat{b}+\omega^{*}\times J\omega^{*}$ . After evaluating initial efficacy, effectiveness against realistically varying systems with noise is investigated.

### 2.10 Noisy Mixed Sensors and Parameter Variations

With the inclusion of feedback, noise must be accounted for in the feedback signals (state and rate only here) in the form of random numbers with a standard deviation of 0.01 (1% of the final state value when states are scaled and balanced to unity). In addition, mass and mass moments are assumed to be unknown or not precisely known; therefore, mass and moments were allowed to vary randomly (uniformly) ten percent heavier and lighter. The resulting scatterplots are presented in section 3.

### 2.11 Scaling and Balancing

Poorly conditioned problems are those requiring simultaneous mathematical operations on very large and small numbers. A common mitigation strategy is to scale and balance the variables transforming equations to nominally remain of the same order. Scaling problems by common, well-known values permits single developments to be broadly applied to a wide range of state spaces not initially intended. Normalizing time per Eq. 20 restricts simulation time to vary between zero and unity. Scaling mass and mass moments of inertia matrices by their nominal values per Eqs. 21 and 22, respectively, keep their values roughly on the order of unity. Generic displacements (translation or rotation) are normalized in accordance with Eq. 23, where {r} could indicate either general translational or rotational displacement.

$$t\equiv \frac{\bar{t}}{t_{f}},$$ (21)

$$\bar{\bar{m}}\equiv \frac{\left[m\right]}{{\left[m\right]}_{nominal}},$$ (22)

$$\bar{\bar{J}}\equiv \frac{J}{J_{nominal}},$$ (23)

$$\bar{r}\equiv \frac{\left\{r\right\}}{{\left\{r\right\}}_{f}}\;for\;translatoin\;and\;\bar{\theta }\equiv \frac{\left\{\theta \right\}}{{\left\{\theta \right\}}_{f}}\;for\;rotation.$$ (24)

## 3 Results

Following the brief introduction to each control technique presented earlier in section 2, section 3 displays the results of individual simulations in addition to the Monte Carlo investigation of ten-thousand simulations. Section 3.1 begins with commonly simplifying assumptions of control design using dominant, double-integrator dynamics with no transport theorem, where the control ${\bar{u}}^{*}\equiv \hat{a}t+\hat{b}$ is applied to the same idealized system equations. The use of idealized results provides interesting measures of performance under ideal circumstances subject to mathematical optimization. Another interesting artifact is the immediately obvious differences in the responses to disparate control techniques.

Next, in section 3.2, the performance of controllers designed using simplified double-integrators ${\bar{u}}^{*}\equiv \hat{a}t+\hat{b}$ was applied to more realistic plant equations with transport theorem. Then, in section 3.3, nonlinear control designs ${\text{u}}_{\text{total}}^{\text{*}}=\hat{a}t+\hat{b}+{\bar{\omega }}^{*}\times \bar{\bar{J}}{\bar{\omega }}^{*}$ are introduced, and comparisons are made applied to nonlinear plants, including transport theorem. Lastly, in section 3.4, random uniformly varying inertia was studied with random noise added to sensor data for both state and rate, and the comparisons were repeated in ten-thousand simulations. These final simulations all utilized nonlinear control designs based on various optimization methods, and the results were applied to nonlinear, coupled system equations, including the transport theorem, where controls were tailored specifically for the transport theorem in the recommended application of optimization (real-time optimal control with singular switching and transport theorem decoupling). All simulations were executed in MATLAB^®^/SIMULINK^®^ R2021b (9.11.0.1769968), whose machine precision was $eps=2.2204\times 10^{-16}$ .

A new presentation style is offered to increase the ease of reading and contemplation of the results. Quantitative figures of merit are presented in tables inserted as sub-figures immediately proximal to corresponding data plots presenting qualitative results.

### 3.1 Ideal, Linear Double Integrator System Equations

**FIGURE 3:**
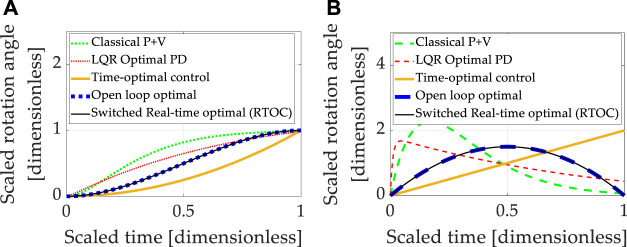
Double-integrator plant (no transport theorem) with control design based on double-integrator. **(A)** Motion *states* (translation or rotational) normalized to propagate from zero and unity in one normalized second. **(B)** Motion *rates* (translation or rotational) intended to propagate from zero initial velocity to zero velocity at the endpoint in one normalized second.

Double-integrator equations expressing relationships between displacement, displacement rate, and acceleration are canonical relationships used to describe the movement of mass. The relationships are linear, allowing easy control design using classical methods, which predominantly use linear systems methods to design controllers for any equations (including linearizing any nonlinear equations). Each control design technique introduced in section 2 was sequentially used to control the linear, double-integrator system equations, and the results are presented in Figure 3, where Table 9 contains quantitative results corresponding to the qualitative results presented in the multi-plots.

**TABLE 9:** Double-integrator plant (no transport theorem) with control design based off double-integrator (no transport theorem): quantitative comparative data corresponding to the qualitative display in Figures 3A,B.

Method	State error	Rate error	Cost	Runtime
Classical *p* + V	0.010115	0.066169	28.1671	3.1012
LQR Optimal PD	0.015015	0.43861	76.3418	2.4597
Time-optimal control	eps	2	2	2.9038
Open loop optimal^a^	eps	Eps	6	2.6086
Real-time optimal (RTOC)^a^	$-9.1882\times 10^{-6}$	0.019289	6.7656	2.7497
Switched RTOC^a^	eps	Eps	6	2.7281

a Real-time optimal control ${\bar{u}}^{*}\equiv \hat{a}t+\hat{b}$ (with and without switching) and open-loop optimal control are visually indistinguishable from one another in the graphic depiction.

The baseline approach (classical proportional plus velocity, or so-called “P + V” control) tuned to performance specification exhibits better accuracy and lower costs than linear-quadratic optimal regulators of the proportional, derivative (PD) type, but the P + V controller has the highest computational burden as indicated by computational runtime. The embedded differentiation of the noisy feedback signal in the rate channel would logically explain the relatively lower performance of the LQR tracking. Time-optimal (bang-bang) control achieved machine precision state tracking accuracy with the largest rate tracking error of the controllers investigated. Such performance is validated by the instinct that time-optimal control is mathematically designed to achieve the desired state in the shortest time but is not structured to simultaneously achieve rate tracking in minimal time. The cost was the lowest of the controllers investigated, indicating the benefits of not requiring simultaneous rate tracking. Computational runtime was the second largest.

Open-loop optimization calculates the minimum control effort that simultaneously meets state and rate endpoint conditions, and accordingly, both state and rate endpoints are achieved to machine precision, while the computational burden is modest compared to low and high cases. Real-time optimization solves the open-loop optimal control problem in real-time using ideal sensor feedback of state and rate but involves a matrix inverse. Rate and state errors (particularly) are quite small, but machine precision tracking is not achieved. Part of the cause of tracking errors is the inversion of the rank-deficient matrix as the final time is approached. Seeking to ameliorate the issue, switched real-time optimal control is presented where the matrix condition is used to switch away from real-time optimal control to open-loop optimal control during timesteps when matrix inversion becomes poorly conditioned. Machine precision tracking is attained, open-loop optimally low costs are re-achieved, and the computational burden is slightly elevated compared to the best case investigated.

Summarizing the results so far, real-time optimal control (designed only to minimize control effort) with singular switching to counter the deleterious effects of poor matrix conditioning achieves the best simultaneous state and rate error (machine precision) with costs matching the open-loop minimal and average computational burden. Unfortunately, these results are achievable only in idealized circumstances of double-integrators. Expressing motion in coordinates of rotating reference frames introduced nonlinear coupling described in the next section (3.2).

### 3.2 Nonlinear Plants With Cross-Product Coupled Transport Theorem With Linear Control Designs

**FIGURE 4:**
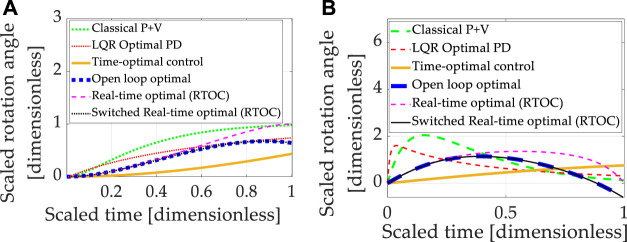
Double-integrator plant (no transport theorem) with control design based on double-integrator. **(A)** Motion *states* (translation or rotational) normalized to propagate from zero and unity in one normalized second. **(B)** Motion *rates* (translation or rotational) intended to propagate from zero initial velocity to zero velocity at the endpoint in one normalized second.

Expressing motion in coordinates of rotating reference frames is referred to as the “transport theorem,” which introduces nonlinear coupling between the six channels of motion that would otherwise have been well-described by simple, linear double-integrators. Very often, linearized system equations or linear assumptions (the double-integrators) are used to design linear controllers. Accordingly, each instance investigated in section 3.1 was applied to nonlinear coupled system equations, including the transport theorem. Increased errors and reduced robustness is generally anticipated since the controllers are not designed to accommodate system nonlinearities specifically. Each control technique introduced in Section 2 was sequentially simulated, and the results are presented in Figure 4, where Table 10 contains quantitative results corresponding to the qualitative results presented in the multi-plots.

**TABLE 10:** Double-integrator plant (*with* transport theorem) with control design based off double-integrator (*without* transport theorem): displays the quantitative comparative data corresponding to the qualitative display in Figure 4.

Method	State error	Rate error	Cost	Runtime
Classical *p* + V	0.024582	0.12803	26.6076	3.4291
LQR Optimal PD	0.26241	0.31372	75.8051	3.4593
Time-optimal control	0.56622	0.76159	0.5	3.4437
Open loop optimal^a^	0.3606	−0.63176	6	3.5315
Real-time optimal (RTOC)^a^	$-1.9654\times 10^{-5}$	0.041323	11.2002	3.4579
Switched RTOC^a^	0.3606	−0.63176	6	3.5097

a Real-time optimal control ${\text{u}}_{\text{total}}^{\text{*}}=\hat{a}t+\hat{b}$ (with and without switching) and open-loop optimal control are visually indistinguishable from one another in the graphic depiction. Notice the control design did not account for nonlinear transport theorem with $u_{\text{nonlinear}}={\bar{\omega }}^{*}\times \bar{\bar{J}}{\bar{\omega }}^{*}$ , the resulting open-loop optimal costs were unchanged, but the state and rate errors increased substantially due to the nonlinear plant not being included in control design. Figure 5 displays the results of including nonlinear control design $u_{\text{nonlinear}}={\bar{\omega }}^{*}\times \bar{\bar{J}}{\bar{\omega }}^{*}$ .

It should be noted that all approaches designed to control double-integrators illustrate degraded performance compared to the idealized case investigated in Section 3.1. All methods compared achieved similar costs. The baseline approach (classical proportional plus velocity, or P + V) tuned to performance specification exhibits second-best state accuracy and second-best rate accuracy, while real-time optimal control achieved the lowest state and rate errors but used nearly double the amount of control. No technique achieved machine precision tracking.

Summarizing the results so far, all control techniques are degraded from the idealized case. Real-time optimal control (designed only to minimize control effort) and classical control methods were the most robust, but all three methods utilized substantially more control effort.

### 3.3 Nonlinear Plants With Cross-Product Coupled Transport Theorem and Nonlinear Control Designs

Double-integrator relationships (implemented identically as done in sections 3.1 and 3.2) are next augmented with feedback decoupling of the transport theorem using state feedback in Eq. 19. Each control design technique introduced in section 2 was sequentially used to control the nonlinear, double-integrator system equations including transport theorem, and the results are presented in Figure 5, where Table 11 contains quantitative results corresponding to the qualitative results presented in the multi-plots.

**FIGURE 5:**
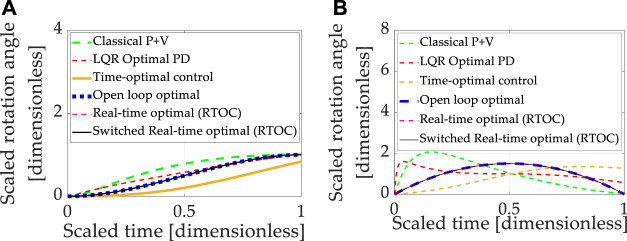
Double-integrator plant (no transport theorem) with control design based on double-integrator. **(A)** Motion *states* (translation or rotational) normalized to propagate from zero and unity in one normalized second. **(B)** Motion *rates* (translation or rotational) intended to propagate from zero initial velocity to zero velocity at the endpoint in one normalized second.

**TABLE 11:** Double-integrator plant (*with* transport theorem) with control design based off double-integrator *with* transport theorem: displays the quantitative comparative data corresponding to the qualitative display in Figures 5A, B.

Method	State error	Rate error	Cost	Runtime
Classical *p* + V	0.0078728	0.038016	27.0064	3.5535
LQR Optimal PD	−0.0063144	0.57121	75.7706	3.5738
Time-optimal control	0.16359	1.2712	2.7286	3.629
Open loop optimal^a^	$3.0287\times 10^{-13}$	$-1.0092\times 10^{-12}$	7.0286	3.4504
Real-time optimal (RTOC)^a^	$-9.1882\times 10^{-6}$	0.019288	7.7942	3.6725
Switched RTOC^a^	$3.0287\times 10^{-13}$	$-1.0092\times 10^{-12}$	7.0286	3.5765

a Unlike the results of Figure 4 where only linear, time-varying control designs ${\text{u}}_{\text{total}}^{\text{*}}=\hat{a}t+\hat{b}$ were used, in Figure 5 nonlinear designs for real-time optimal control ${\text{u}}_{\text{total}}^{\text{*}}=\hat{a}t+\hat{b}+{\bar{\omega }}^{*}\times \bar{\bar{J}}{\bar{\omega }}^{*}$ (with and without switching) were used. Notice the open-loop optimal control is visually indistinguishable from nonlinear, time-varying control designs in the graphic depiction.

All methods are improved by addition of the nonlinear decoupling control designed for the transport theorem. Time-optimal control performs worst regarding state and rate errors, while cost figures are generally increased for methods that effectively track state and rate. Near machine-precision is achieved by open-loop optimal control and switched, real-time optimal control where both are designed to minimize control effort alone (with no state error representation in the minimized cost function). Computational burdens of all approaches are roughly comparable.

Having initially analyzed idealized systems (the double-integrators), nonlinear coupling was induced by the transport theorem with significantly degraded performance using the controllers designed for linear systems. Adding nonlinear control components designed specifically to decouple the transport theorem in feedback roughly restores nominal performances, but feedback remains ideal (without noise). Section 3.4 adds zero-mean Gaussian noise to both sensor types (state and rate)

### 3.4 Nonlinear Plants With Cross-Product Coupled Transport Theorem and Nonlinear Control Designs Utilizing Noisy, Mixed-Sensors

**FIGURE 6:**
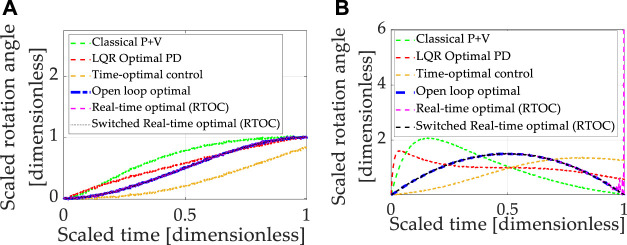
Double-integrator plant (with transport theorem) with control design based on double-integrator (with transport theorem). **(A)** Motion *states* (translation or rotational) normalized to propagate from zero and unity in one normalized second. **(B)** Motion *rates* (translation or rotational) intended to propagate from zero initial velocity to zero velocity at the endpoint in one normalized second. Notice that the open-loop optimal (minimum) control effort is increased from 6.0 (controlling double-integrators without transport theorem) to just over 7.0 (controlling double-integrators with transport theorem), manifesting as a 17% increase to account for transport theorem over idealized cases of double-integrators alone.

Double-integrator equations with nonlinearities induced by transport theorem were controlled by linear control designs augmented with nonlinear feedback decoupling designed specifically for transport theorem. Feedback was provided by simulated mixed state and rate sensors, and Gaussian noise was added. Each control design technique introduced in section 2 was sequentially used to control the linear, double-integrator system equations, and the results are presented in Figure 6, where Table 12 contains quantitative results corresponding to the qualitative results presented in the multi-plots.

**TABLE 12:** Double-integrator plant (*with* transport theorem) with control design based off double-integrator *with* transport theorem and noisy, mixed sensors (state and rate): sub-displays the quantitative comparative data corresponding to the qualitative display in sub-Figure 6.

Method	State error	Rate error	Cost	Runtime
Classical *p* + V	−0.0066807	0.03828	27.0755	2.3988
LQR Optimal PD	−0.0047636	0.56965	76.8806	2.4667
Time-optimal control	0.16546	1.2693	2.7286	2.4837
Open loop optimal^a^	0.0018665	−0.0018665	7.0286	2.5816
Real-time optimal (RTOC)^a^	0.06463	−171.6553	41,436,948	2.469
Switched RTOC^a^	0.0018665	−0.0018665	7.0286	2.6125

a Real-time optimal control ${\text{u}}_{\text{total}}^{\text{*}}=\hat{a}t+\hat{b}+{\bar{\omega }}^{*}\times \bar{\bar{J}}{\bar{\omega }}^{*}$ (with and without switching) and open-loop optimal control are visually indistinguishable from one another in the graphic depiction.

The baseline approach (classical proportional plus velocity, or P + V) tuned to performance specification exhibits better rate accuracy and lower costs than linear-quadratic optimal regulators of the proportional, derivative (PD) type, but the P + V controller has relatively inferior state errors compared to LQR. Time-optimal control performs poorly in the face of transport theorem and noisy sensors. Real-time optimal control is severely degraded by noise, particularly with respect to rate errors and control effort.

Open-loop optimization and real-time optimal control with singular switching simultaneously achieve the lowest state and rate endpoint errors, with the lowest costs (that may be claimed to meet endpoint conditions), while the computational burden is modest compared to low and high cases.

#### 3.4.1 Monte Carlos Analysis (6,000 Simulation Runs)

Summarizing the results so far, real-time optimal control (designed only to minimize control effort) with singular switching to counter the deleterious effects of poor matrix conditioning achieves the best simultaneous state and rate error with costs matching the open-loop minimal and average computational burden in cases where nonlinear feedback decoupling of transport theorem is incorporated and where feedback is provided by noisy state and rate sensors.

## 4 Discussion

The results are multi-variate, but some general comments are evident regarding the proposed real-time optimal control with singular switching and transport theorem decoupling and its performance compared to a classical benchmark and four other instantiations of optimal control. In the most realistic situations revealed by Monte Carlo analysis with random variations of inertia and state and rate sensor noise, time-optimal bang-bang control achieved respectable rate accuracy with the lowest cost but highest runtime and modest rate tracking errors. Meanwhile, optimal (control minimizing constrained to meet endpoint conditions) open-loop control and its companion real-time optimal control with singular switching achieved the lowest state errors (three orders of magnitude better than time-optimal control) and control effort, while real-time optimal control with singular switching and transport theorem decoupling achieved the lowest rate tracking error. Real-time optimal control without singular switching displayed vulnerability in rate errors and high costs.

Other general conclusions apply to all techniques: designing controls based on simplified plants and then applying them to realistic plants is particularly weak compared to the relatively modern approaches. Arguably, milli-degree accuracy with “low” costs is admirable performance, but the modern methods of control design, including optimality and nonlinear coupling effects (with feedback), achieved, in general, three orders of magnitude superior performance, with the admission that real-time optimal control performed particularly poorly.

Furthermore, well-known lessons from classical control are re-validated in this study. Linear-quadratic regulators are very robust and useful, but suffer from cascaded topologies, particularly in the differentiation of the state feedback to achieve rate feedback, thus the utilization of velocity control was established as the classical baseline (with a requisite demand to purchase and utilize rate sensors).

The proposed instantiation of real-time optimal control with singular switching and nonlinear transport theorem decoupling ${\text{u}}_{\text{total}}^{\text{*}}=\hat{a}t+\hat{b}+{\bar{\omega }}^{*}\times \bar{\bar{J}}{\bar{\omega }}^{*}$ was the overall top-performing option with the lowest state errors, lowest rate errors, lowest computational burden, and second-lowest control effort (fuel usage).

Lastly, it should be noted that all the control techniques performed very well (naturally, since most of the techniques were formulated to satisfy optimization problems). The indication of superior performance should not be judged as mandating the proposed technique, especially in instances where operators would be more comfortable with classical techniques and the order of milli-degree accuracy is sufficient.

### 4.1 Performance Improvement Percentages

The claim was just immediately earlier, validating that real-time optimal control with singular switching and transport theorem decoupling was the overall top-performing option, and this section describes the results validating the claim in generally understandable terms (percent performance improvement comparison). Open-loop (control minimizing optimal control constrained to meet end state and rate) performed very well, while real-time optimal control with singular switching matched the performance and was slightly better in terms of computational burden.

### 4.2 Future Research

The derivation of optimal trajectories (state, rate, acceleration, and jerk) should prove useful in the implementation of deterministic artificial intelligence (Smeresky et al., 2020), which requires some scheme of autonomous trajectory generation. The current state of the art utilizes sinusoidal trajectory generation schemes, and the optimal trajectories illustrated here should have improved efficacy when used to augment deterministic artificial intelligence.

## 5 Conclusion

Real-time optimal control is proposed to deal with nonlinear mechanics, including transport theorem coupling nonlinearities, where noisy (random) sensors are assumed, and random parameter variation is countered with time-varying solutions to Pontryagin’s necessary conditions of optimality. Specifically, the Hamiltonian minimization condition and the adjoint equations produce the form of the control parameterized in terms of time and mass or mass moment of inertia, respectively. Singularity-based switching is proposed to address divergence of the adjoints approaching the final state. Ubiquitous figures of merit are used to compare the proposed methods to benchmark classical and modern optimal control methods: mean state and rate errors, quadratic costs embodying necessary fuel usage, and computational runtime as an avatar of the computational burden. Open-loop optimal control established an intermediate baseline over the benchmark classical control, while the proposed method yielded identical performance improvements in terms of state and rate accuracy and quadratic cost while experimentally illustrating an unexpected ten percent improvement in computational burden.

## Data Availability

The raw data supporting the conclusions of this article will be made available by the authors, without undue reservation.
