# Supplementary material for: Treatise on Analytic Nonlinear Optimal Guidance and Control Amplification of Strictly Analytic (Non-Numerical) Methods
Source: Front Robot AI. 2022 Oct 4;9:884669. doi: 10.3389/frobt.2022.884669 (PMC9583946; doi:10.3389/frobt.2022.884669)
Supplement: Supplementary file 1 [file DataSheet1.docx]

**Appendix A**

The appendix contains details of the simulations, first with SIMULINK topologies, and secondly with MATLAB line-code to aid the readership replicate the experiments presented in this manuscript.

|  |  (**b**)   |
| --- | --- |
| (**a**) | (**c**) |

**Figure 6.** SIMULINK topologies used to produce the results in section 3. The controllers subsystem is detailed in figure 7. Sub-figure (a) displays the overarching topology, (b) double integrator dynamics with and without transport theorem (in red for added emphasis) with a selectable switch, (c) sensor subsystem adding random noise to both state and rate.

**Figure 7.** SIMULINK model for controllers where manual switches are used to evaluate each technique in direct comparison where all other facets are held constant except the control methodology used. The coloring of the blocks in the figure are consistent with the coloring of the data lines plotted in subsequent figures throughout section 3 of the manuscript. Red coloring indicates linear-quadratic regulator with proportional derivative control, orange indicates time-optimal control. Green color indicates proportional plus velocity (P+V) control. Pink indicates real-time optimal control, blue indicates open loop optimal control, and black indicates transport theorem control components.

**Figure 8**. SIMULINK model implementing real-time optimal control with and without singular switching (manually selectable using the switch on the right-hand side of the simulation graphic). The red simulation block labeled “Invert [T]” is displayed in figure 9.

**Figure 9.** Simulation blocks implementing options for matrix inversion in real-time optimal control.

clear all; close all; clc; warning off;

ENDSTATE=[]; COSTEND=[]; TICTOC=[]; COST=[];

for i=1:1000; tic; i

sim('IterateOnOptimalControl'); toc;

EndState=States(end,:); ENDSTATE=[ENDSTATE; EndState];

COSTEND=[COSTEND; Cost(end)]; TICTOC=[TICTOC; toc];

COST=[COST; squeeze(Cost)];

end

C = [1-mean(ENDSTATE(:,1)) mean(ENDSTATE(:,2))] ; % ellipse center

a = std(1-ENDSTATE(:,1)); b = std(ENDSTATE(:,2)); % major and minor axis

hold on; plot(C(1)+a*cos(linspace(0,2*pi)),C(2)+b*sin(linspace(0,2*pi)) ,'k') ;

plot(C(1)+2*a*cos(linspace(0,2*pi)),C(2)+2*b*sin(linspace(0,2*pi)) ,'k') ;

plot(C(1)+3*a*cos(linspace(0,2*pi)),C(2)+3*b*sin(linspace(0,2*pi)),'k') ;

scatter(1-ENDSTATE(:,1), ENDSTATE(:,2)); grid; axis equal;

set(gca,'fontsize',18, 'fontname','palatino linotype');

LEGENDTEXT1=['\mu TicToc = ' num2str(mean(TICTOC))]

LEGENDTEXT2=['; \mu StateError = ' num2str(1-mean(ENDSTATE(:,1)))]

LEGENDTEXT3=['; \mu RateError = ' num2str(mean(ENDSTATE(:,2)))]

LEGENDTEXT4=['; \mu Cost = ' num2str(mean(COST))]

LEGENDTEXT=[LEGENDTEXT1 LEGENDTEXT2 LEGENDTEXT3 LEGENDTEXT4]

legend(LEGENDTEXT)

hold off; clc;
